# Lack of microbiological awareness on the ward as a key factor for inappropriate use of anti-infectives: results of a point prevalence study and user satisfaction survey in a large university hospital in Austria

**DOI:** 10.1007/s15010-023-02150-4

**Published:** 2023-12-27

**Authors:** Jan Marco Kern, Karoline Berger, Arno Michael Lechner, Ulrike Porsche, Markus Wallner, Eva Maria Past

**Affiliations:** 1 Institute of Clinical Microbiology and Hygiene, Paracelsus Medical Private University, University Hospital Salzburg, Muellner Hauptstrasse 48, 5020 Salzburg, Austria; 2 Department of Clinical Pharmacy and Drug Information, State Pharmacy, University Hospital Salzburg, Muellner Hauptstrasse 50, 5020 Salzburg, Austria

**Keywords:** Antimicrobial stewardship, Diagnostic stewardship, Antimicrobial treatment, Microbiology

## Abstract

**Purpose:**

Although diagnostic stewardship issues in clinical microbiology harbor an optimization potential for anti-infective consumption, they are only marginally addressed in antimicrobial stewardship (AMS) programs. As part of an AMS point prevalence (PPS) survey we therefore aimed to gain a more dynamic view on the microbiological awareness within therapeutic regimens. By examining whether initial microbiological sampling was performed and in which way microbiological results were incorporated into further treatment considerations we sought to find out to what extent these points determine the appropriateness of treatment regimens.

**Methods:**

PPS was performed at the University Hospital Salzburg (1524 beds) in May 2021. Relevant data was determined from the patient charts and the appropriateness of anti-infective use was assessed using predefined quality indicators. Six months after the PPS, a questionnaire was administered to clinicians to obtain information on the use of microbiological findings and their relevance in the clinic.

**Results:**

Lack of microbiological awareness in the clinical setting proved to be the key reason for an overall inadequate use of anti-infectives (35.4% of cases rated as inadequate), ahead of the aspects of dose (24.1%), empirical therapy (20.3%) and treatment duration (20.2%). This was particularly the case for broad-acting agents and was most evident in urinary tract infections, skin and soft tissue infections, and pneumonia. The results of the questionnaire indicate a discrepancy between the physicians surveyed and the routine clinical setting.

**Conclusion:**

A high potential in improving the use of anti-infectives in hospitals seems to lie in a strong emphasis on microbiological diagnostic stewardship measures.

## Introduction

The global increase in microorganisms that are resistant to anti-infectives poses a major threat to our healthcare systems [1]. Multidisciplinary antimicrobial stewardship (AMS) programs, in particular, have become the cornerstone of counterstrategies for optimizing anti-infective use and combating the development of anti-infective resistance to improve patient outcomes [2–4]. Diagnostic stewardship (DSS) has just recently emerged as a sub-discipline within AMS strategies and the microbiology laboratory can play a key role here as—beyond its diagnostic focus—it is involved in essential pre- and post-analytical components of antimicrobial management [5–7]. However, AMS measures are only valuable if their implementation is regularly evaluated to assess the entire AMS effort with the aim of uncovering corresponding weaknesses in the system. In this way, targeted improvements can be made by the AMS team on site.

The current study sought to evaluate the local AMS program by a point prevalence survey (PPS) in a tertiary hospital. On the one hand, we wanted to observe classic AMS issues of antimicrobial use such as indication and anti-infective spectrum, dose, administration interval, and duration of treatment. Our major topic, however, was to assess the pre- and post-analytical components of microbiological DSS, which are frequently overlooked in traditional AMS surveys. To highlight a potential impact of microbiological DSS on AMS strategies, we evaluated both the performance of initial sample collection (pre-analytical phase) and whether and how microbiological findings are incorporated into further treatment considerations (post-analytical phase), with the goal of evaluating potential improvement strategies related to antibiotic use.

In addition, the AMS team conducted a user satisfaction survey via an online questionnaire 6 months after the implementation of the PPS to assess clinicians’ user behavior regarding the application of local therapy recommendations and their satisfaction with microbiological results in terms of quality, presentation of findings, and clinical applicability of microbiological results.

## Methods

### Point prevalence survey

This PPS was carried out at the University Hospital Salzburg which offers the full spectrum of tertiary care on two campuses in the city of Salzburg: CDK campus (Christian-Doppler-Klinik, 484 inpatient beds) with a focus on neurology, neurosurgery and psychiatry, and the LKH campus (Landeskrankenhaus, 1 040 inpatient beds). The audit was performed on 1 day in May 2021 by eight members of the AMS team in pairs. Each pair consisted of a pharmacist and a physician (infectious diseases, clinical microbiology or infection control) or an infection control nurse. The head of each department and the chief nurse were informed about the PPS but the exact date remained unknown until the day of the PPS.

### Inclusion criteria of patients for the PPS

All inpatients who physically were on the ward during the time of the PPS with at least one active prescription of an antiinfective drug (oral or parenteral route) for prophylactic or therapeutic purposes was included into the study. Patients were excluded if the antiinfective drug was applied topically.

### Data of interest and failure categories

Patient data was extracted from the medical patient records (paper-based or electronic medical record (ORBIS^®^/ORME^®^ (Dedalus) or MetaVision^®^ (iMDSoft)) and a specially designed audit form was completed. The focus was on patient-related information, antimicrobial agents (dosage, route of administration, current treatment duration), a clearly documented diagnosis and focus of infection with the indicated purpose of treatment as well pre- and post-analytical microbiologic aspects (state of initially performed sample collection, implementation of results into further treatment considerations) (Table 1). Subsequently, predefined failure indicators could be derived on the basis of the infectious entity and standard regimens (treatment and prophylaxis) according to national and international guidelines, as well as the current AMS recommendations of the hospital (Table 2). In the event of at least one positive failure category, the case was addressed “inadequately”. In case of poor or incomplete documentation of the patient record, the case was categorized “indeterminable”.

**Table 1:** Data of interest

Main aspect	Data of interest
Patient-related aspects	Age
	Sex
	Body height, body weight
	Renal function
	Documented hypersensitivity towards antimicrobial agents
Antimicrobial agents	Substance
	Dosage
	Route of administration (iv/ po/ other)
	Duration of administration to date
Indication	Documented diagnosis
	Focus of infection
	Purpose of treatment (empirically/ targeted/ prophylactic)
	In case of prophylaxis: perioperative/ other
Microbiology	Sample collection (yes/no)
	Result-driven treatment or treatment adaption (yes/ no)

**Table 2:** Failure indicators

Main aspect	Failure indicators
Spectrum of empiric treatment	Narrow—adequate—broad
Dosage	Low—adequate—high
Duration of treatment	Lack of evaluation—adequate
	Unnecessary duration of perioperative prophylaxis
Microbiology	Lack of implementation of microbiologic results
	Lack of initial sample collection
Clinical focus	Lack of documented indication of antimicrobial treatment

### Data entry and statistical analysis

Data entry into MS-Excel^®^ (Microsoft) was performed by two typists from the secretary pool of the university hospital. Descriptive data analysis was performed in MS-Excel^®^ (Microsoft) by the AMS team.

### User satisfaction survey

Six months after the PPS, the AMS team conducted a user satisfaction survey as a follow-up. An online questionnaire was distributed to all clinicians asking about the degree of satisfaction with the AMS team’s advising function as well as the quality and applicability of microbiologic results. A member of the quality management team evaluated the questionnaires and processed the data (Table 3).

**Table 3:** Questionnaire aspects

No	Demography of clinicians	Queried aspects
1	Degree of medical education	Basic physician Assistant physician Specialist Senior physician or higher
2	Years of service	< 5 5–10 11–15 16–20 > 20
3	Knowledge about the department schemas	Yes/no
4	Knowledge about the clinic-wide schemas	Yes/no
5	Frequency of use of the department scheme for the empiric phase of antiinfective treatment	Range 1–5 1—always 5—never
6	Frequency of use of the clinic-wide schemes	Range 1–5 1—always 5—never
7	Grade of satisfaction with the AMS team - Quality of infectious disease consultation - Quality of pharmaceutical consultation - Duration until the first contact of the AMS team after the order of the consilium - Applicability of the department scheme - Procedure of requesting indexed/ restricted antiinfectives	Range 1–5 1—very satisfied 5—very dissatisfied
8	Grade of satisfaction with microbiologic results - Quality of microbiologic results - Presentation of microbiological findings - Clinical applicability of microbiological findings	Range 1–5 1—very satisfied 5—very dissatisfied
9	Point prevalence survey - Knowledge about the PPS results - Knowledge about detailed PPS results (department level)	Yes/no Yes/no
10	Results of the point prevalence survey Ability to derive significant aspects for therapy management concerning - Microbiological diagnostic - Treatment duration - Dosing interval - Effective antimicrobial spectrum of substances	Range 1–5 1—fully applies 5—does not apply at all
11	Level of matching results with expectations concerning - Lack of implementation of microbiologic results in treatment considerations - Lack of initial sample collection - Treatment duration - Dosing interval - Effective antimicrobial spectrum of substances - Perioperative prophylaxis	Range 1–5 1—fully applies 5—does not apply at all

## Results

### Results from the point prevalence survey

Of the 1021 patient records screened, 24.4% of patients had at least one antimicrobial prescription (*n* = 249). The rate of patients with antimicrobial prescriptions varied from 4.0% in the department of psychiatry to 66.7% in the department of anesthesiology, the latter including three intensive care units. No antimicrobials were prescribed in the child and adolescent psychiatry department (Table 4).

**Table 4:** Rate of patients undergoing antimicrobial treatment

Department	Available patient records (*n*)	Patients with antimicrobial treatment [*n* (%)]
Anesthesiology (incl. ICU)	27	18 (66.7)
Urology	29	17 (58.6)
Internal medicine III^a^	73	36 (49.3)
Pulmonary medicine	32	13 (40.6)
Oro-maxillofacial surgery	10	4 (40.0)
Internal medicine II^b^	94	33 (35.1)
Surgery	56	19 (33.9)
Orthopedics and traumatology	64	18 (28.1)
Dermatology	23	6 (26.1)
Neuro surgery^c^	52	13 (25.0)
Internal medicine I^d^	67	15 (22.4)
Pediatric surgery	19	4 (21.1)
Cardiac surgery (incl. vascular surgery section)	24	5 (20.3)
Geriatrics^c^	91	15 (16.5)
Pediatrics	38	6 (15.8)
Neurology, neurologic ICU^c^	97	13 (13.4)
Otorhinolaryngology	24	3 (12.5)
Radio oncology	20	2 (10.0)
Ophthalmology	22	2 (9.1)
Gynecology	57	4 (7.0)
Psychiatry^c^	75	3 (4.0)
Child and adolescent psychiatry^c^	27	0
Total	1021	249 (24.4)

^a^Hematology, oncology, rheumatology

^b^Cardiology, cardiologic intensive care unit (ICU)

^c^Campus Christian-Doppler-Klinik

^d^Gastroenterology, diabetology, nephrology, hepatology

### Patient characteristics and antimicrobial course

Demographic patient characteristics are displayed in Table 5. Male patients were slightly more represented (55%) compared to female patients (45%) and age distribution showed a median age of 69.5 years. Most patients (*n* = 185, 74.3%) received a single antimicrobial agent, 20.8% of patients (*n* = 52) received two substances, whereas 4.8% (*n* = 12) had a course with three agents. Agents were mainly administered intravenously (80.9%, *n* = 263). The vast majority of patients received an antibiotic (93.8%) followed by antifungals and antivirals (3.4% and 2.8% respectively). No anthelmintic agents were given on the surveyed day.

**Table 5:** Patient characteristics

Parameter	[*n* (%)]
**Sex**	
Female	112 (45.0)
Male	137 (55.0)
Total	249 (100)
**Age (years)**	
0–18	11 (4.4)
19–40	21 (8.4)
41–64	64 (25.7)
65–79	95 (38.2)
80–92	58 (23.3)
**Number of currently administered antimicrobial agents**	
1	185 (74.3)
2	52 (20.9)
3	12 (4.8)
**Specification of antimicrobial agent**	
Antibiotic	305 (93.8)
Antifungal	11 (3.4)
Antiviral	9 (2.8)
Anthelmintic	0
**Documented indication of antimicrobial used**	195 (78.3)
**Route of administration**	
p.o	62 (19.1)
i.v	263 (80.9)

### Reasons for inadequate antimicrobial treatment

**Fig. 1:**
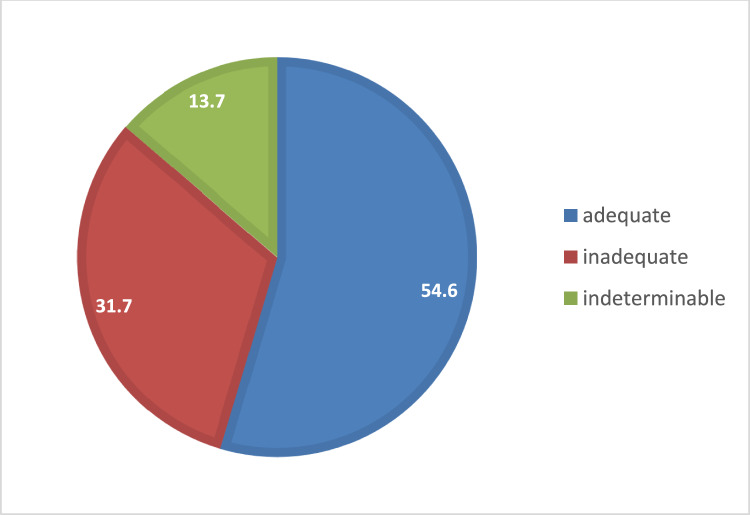
Adequateness of antimicrobial use

With regard to the predefined failure indicators of above mentioned categories (Table 2), in total 54.6% (*n* = 136) of analyzed patients were treated adequately, whereas, in 31.7% of cases (*n* = 79) the patients received an inadequate antimicrobial course (Fig. 1). The rate of indeterminable patient data was 13.7% (*n* = 34).

**Fig. 2:**
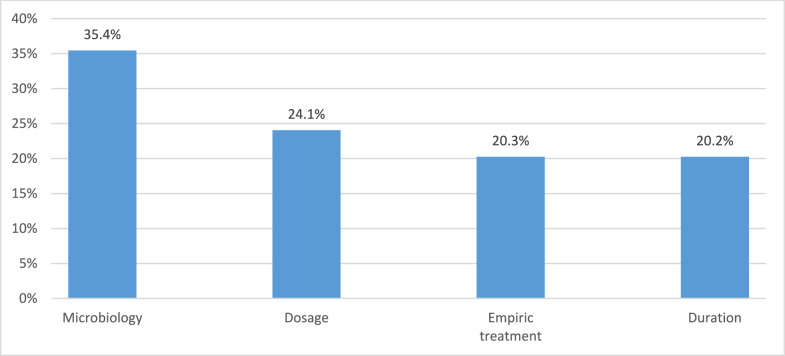
Reasons for inadequate antimicrobial treatment

**Fig. 3:**
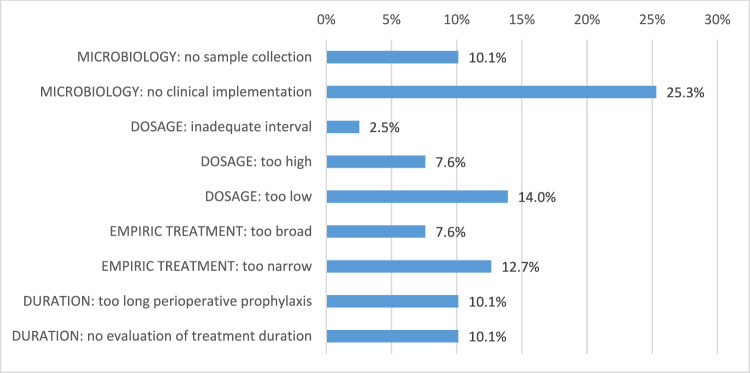
Reasons for inadequate antimicrobial treatment (detailed)

Pre- and post-analytic processes in the category “microbiology” turned out to be the major failure category in this PPS, accounting for 35.4% of inadequate cases. Non-performance of initial sample collection (10.1%) as well as lack of implementation of microbiologic results into further treatment considerations (25.3%) could be depicted as main reasons for an overall inadequate antimicrobial treatment (Figs. 2, 3).

In 24.1% of cases the dosage of the antimicrobial substance in question was inappropriate related to the clinical indication with a dose either a too low (14.0%), too high (7.6%) or an insufficient dosing interval (2.5%). With regard to the diagnose-triggered empirical therapy, we found that a total of 20.3% of cases were inadequately designed with the anti-infective spectrum being either too narrow (12.7%) or too broad (7.6%). A similar high percentage of inadequate therapy duration (20.2%) was observed: In 10.1% of the reported cases, treatment duration up to the study day was either no longer evaluated or an initial perioperative prophylaxis was unreasonably prolonged.

### Role of microbiologic awareness in inappropriate antimicrobial management

**Fig. 4:**
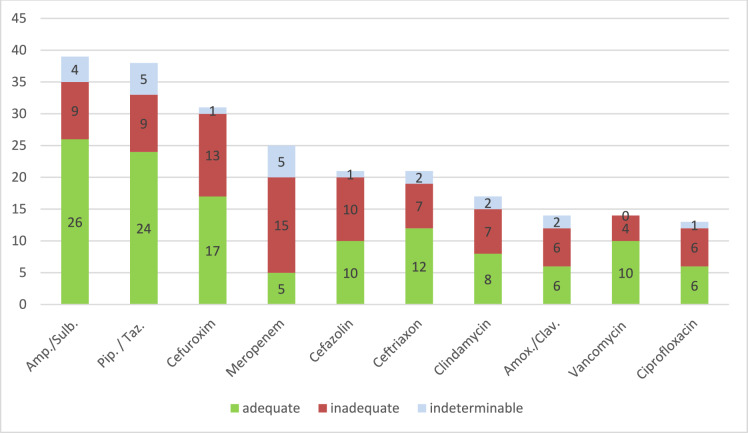
Most prescribed antimicrobial agents. *Amb./Sulb* ampicillin/sulbactam, *Pip./Taz.* piperacillin/tazobactam, *Amox./Clav.* amoxicillin/clavulate

Betalactam antibiotics dominated the list of the most commonly prescribed antibiotics, making up approximately 60% of all antiinfectives, followed by ciprofloxacin and clindamycin (Fig. 4). The by far highest potential for an inadequate course could be depicted for meropenem (Table 6). While treatment with this substance was documented in only 7.7% of all patients, the substance was inadequately administered in 60% of these cases. The main causes of an improper meropenem treatment was a lack of microbiologic awareness in 53.3% of cases, with either a failure to implement existing laboratory results (33.3%), or a failure to collect the initial sample (20%).

**Table 6:** Main reasons for inadequate antimicrobial use

Substance	Inadequate use (%)	Use among all prescriptions (%)	Main reasons for inadequate antimicrobial use
Meropenem	60	7.7	53.3% lack of microbiologic awareness - 33.3% no implementation of microbiologic results - 20.0% no sample collection 39.9% inadequate dosage - 33.3% dosage too low - 6.6% dosage too high 6.6% inadequate interval
Cefazolin	47.6	8	40% inadequate dosage - 30% dosage too high - 10% dosage too low 20% duration too long
Ciprofloxacin	46.2	4	66.6% lack of microbiologic awareness - 50% no implementation of microbiologic results - 16.6% no sample collection 33.3% empiric treatment too broad
Amoxicillin/ Clavulate	42.9	4.3	50% lack of microbiologic awareness (no implementation of microbiologic results) 33.3 inadequate dosage (too low) 17% empiric treatment too broad
Cefuroxime	41.9	9.5	53% prolonged perioperative prophylaxis 38.4% lack of microbiologic awareness - 30.7% no implementation of microbiologic results - 7.7% no sample collection
Clindamycin	41.2	5.2	42.7% lack of microbiologic awareness - 28.5% no implementation of microbiologic results - 14.2% no sample collection 14.2% inadequate dosage (too low) 14.2% empiric treatment too narrow
Ceftriaxone	33.3	6.5	28.6% lack of microbiologic awareness - 14.3% no implementation of microbiologic results - 14.3% no sample collection 28.6% empiric treatment too narrow 14.3% inadequate dosage (too high)
Vancomycin	28.6	4.3	75% inadequate dosage (too high)
Piperacillin/ tazobactam	23.7	11.7	44.4% lack of microbiologic awareness - 33.3% no implementation of microbiologic results - 11.2% no sample collection 22.2% empiric treatment too narrow 11.1% inadequate dosage (too low)
Ampicillin/ sulbactam	23.1	12	55.5% inadequate empiric treatment - 33.3% spectrum too broad - 22.2% spectrum too narrow 22.2% lack of microbiologic awareness - 11.1% lack of microbiologic awareness - 11.1% no sample collection

Comparable data not only for the use of ciprofloxacin (66.6% inadequate use due to lack of microbiologic awareness), amoxicillin/clavulate (50%), and piperacillin/tazobactam (44.4%) but also for clindamycin (42.7%) and cefuroxime (38.4%) could be shown. In terms of inadequate dosage, which was the second relevant aspect, especially cefazolin and vancomycin showed to be particularly important. However, prolonged treatment duration was demonstrated as a relevant failure indicator for cefuroxime and cefazolin, which both are frequently used in perioperative prophylaxis. Table 6 provides a detailed overview of the main reasons for inadequate antiinfective management of the mentioned substances.

**Fig. 5:**
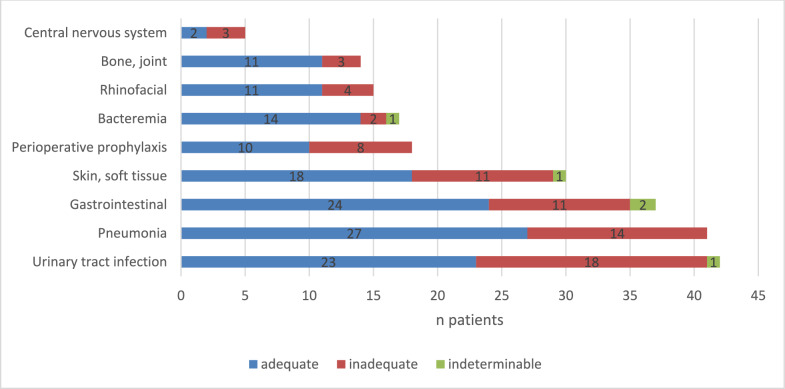
Most relevant infection sites for antimicrobial use. Gastrointestinal = incl. *Clostridium difficile* enterocolitis, pneumonia = incl. noscocomial and community-acquired pneumonia

At the level of the most relevant indications and infectious entities, urinary tract infection (*n* = 42 cases), pneumonia (*n* = 41), infections of the gastrointestinal tract (*n* = 37), skin, and soft tissue infections (*n* = 30) and perioperative prophylaxis (*n* = 18) emerged as the most frequent reasons for anti-infective treatment (Fig. 5). Of these, perioperative prophylaxis and urinary infections showed the highest potential for an overall inadequate use of antiinfectives (44.4% and 42.9% respectively) followed by skin and soft tissue infections (36.7%) and pneumonia (34.1%).

**Fig. 6:**
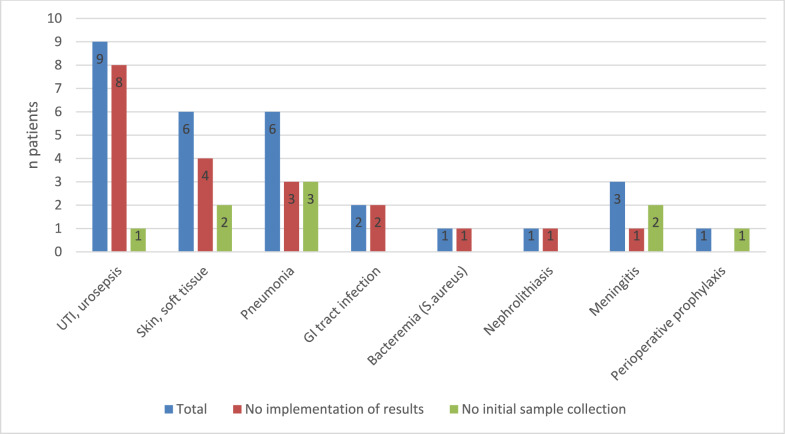
Lack of microbiologic awareness in main infectious entities. UTI: urinary tract infection, GI: gastrointestinal

With particular attention to the microbiology aspects, lack of microbiologic awareness had a detrimental effect on therapeutic management of urinary tract infections (*n* = 9 cases in total). Here, the focus was obviously the absence of integration of microbiologic results into subsequent treatment considerations (*n* = 8 inadequate cases), whereas, initial specimen collection was not an issue (*n* = 1 inadequate case). Comparable findings could be shown for skin and soft tissue infections and pneumonia (*n* = 6 cases each in total), where the antimicrobial treatment was adversely affected by the substantial unawareness considering microbiologic sampling and results (Fig. 6).

### Results from the user satisfaction survey

#### Demography

**Fig. 7:**
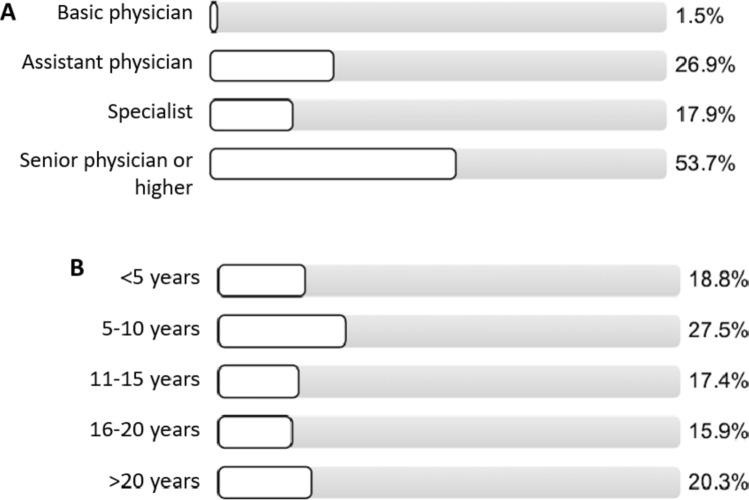
Level of education of clinicians (**A**) and years of experience (**B**)

**Fig. 8:**
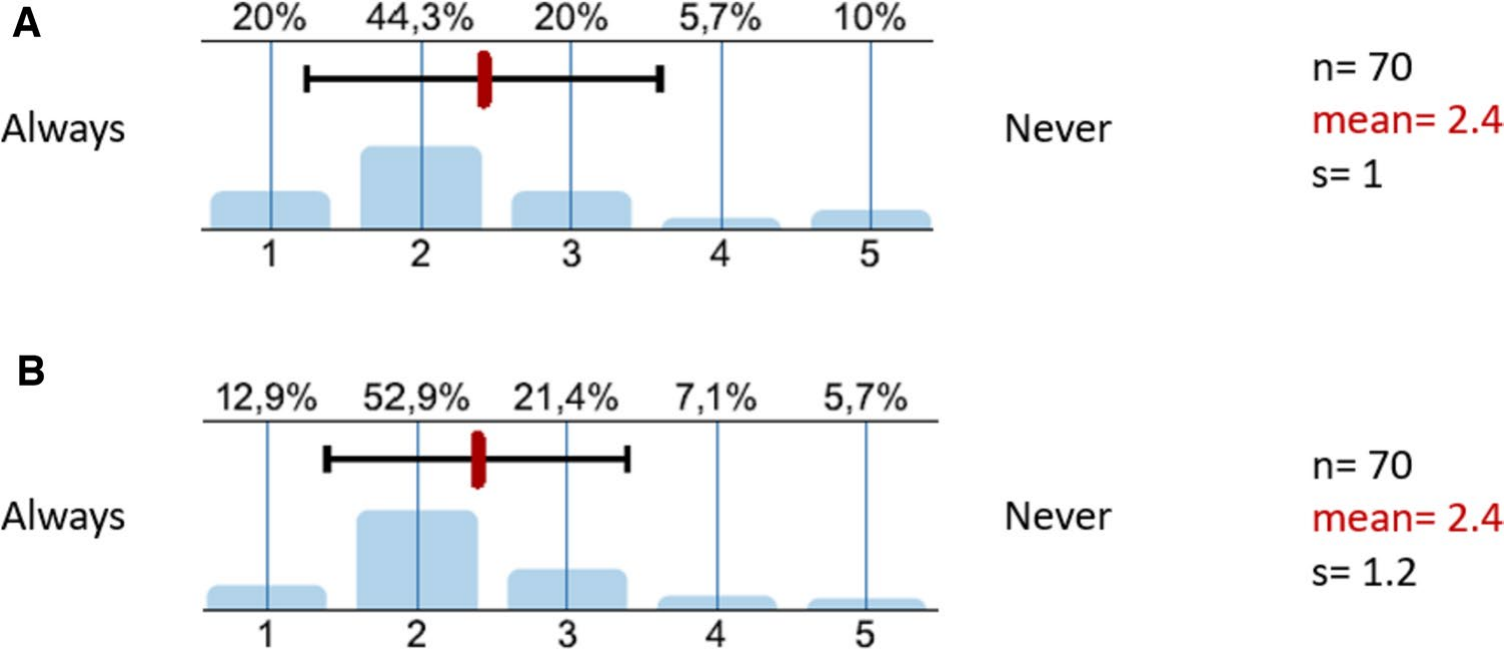
Frequency of application of the clinic-wide (**A**) or the department schemes (**B**). *s* standard deviation

Altogether 71 questionnaires were returned indicating a response rate of 7.1%. The majority of participants (71.6%) were physicians with a high level of education being either specialists, senior physicians or higher compared to 28.4% of physicians in the beginning of their training (Fig. 7A). Most physicians (81.1%) had been at service for at least 5 years with 53.6% of participants with a clinical experience of more than 10 years (Fig. 7B). Concerning the general knowledge of participants about antimicrobial recommendations of the AMS team 87% stated that they were familiar with the clinic-wide schemes, whereas 88.4% of participants reported knowing the department scheme (data not shown). With regard to the frequency of clinical application of either the clinic-wide and the department schema the physicians stated to often resort to the scheme in 64.3% and 65.8% respectively (Fig. 8). The vast majority (98.5%) of participating clinicians stated to be satisfied (value 2 on a scale of 1–5 ranging from very satisfied to very dissatisfied) or very satisfied (value 1) with the infectious diseases consultation provided by the AMS team, indicating a high acceptance of the team in the hospital (data not shown).

**Fig. 9:**
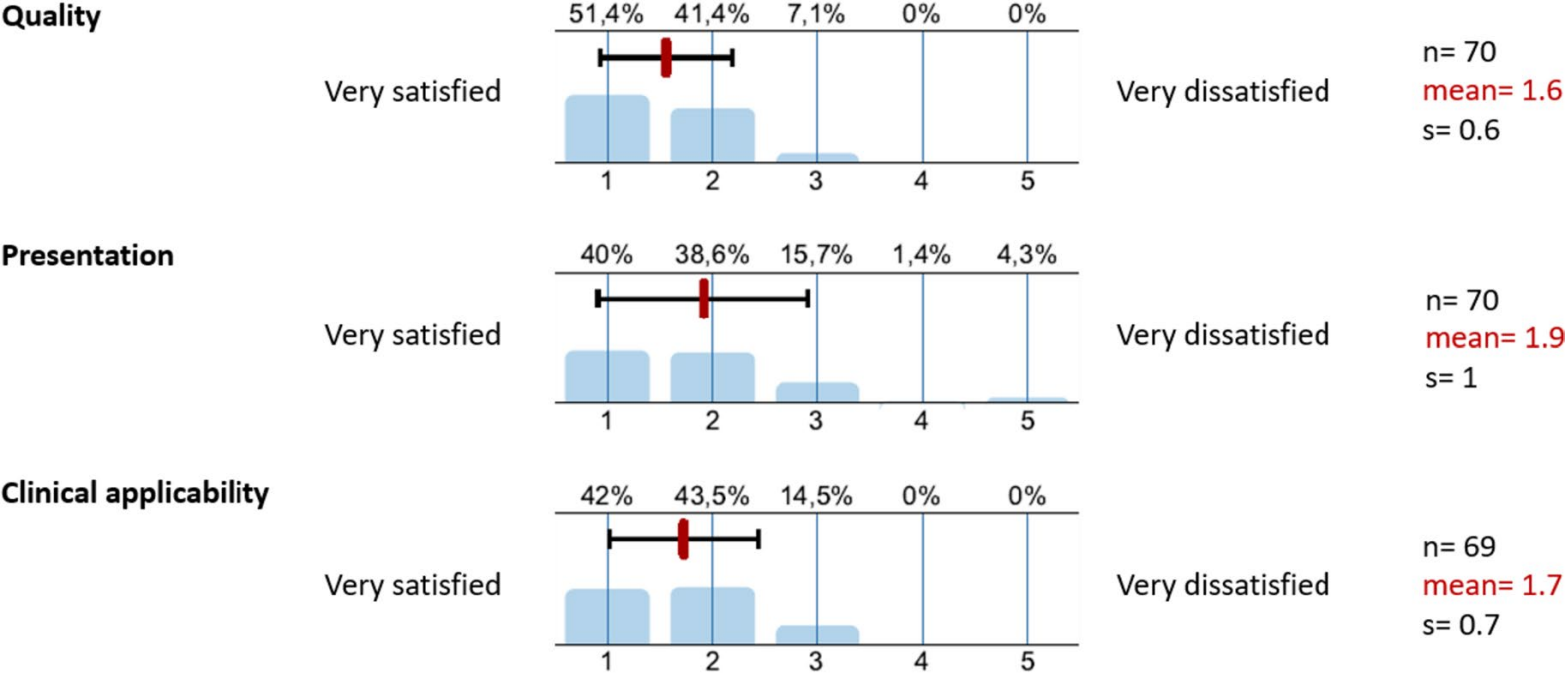
Grade of satisfaction with microbiologic results. *s* standard deviation

The overall satisfaction of the participants regarding microbiological results (Fig. 9) can be considered high to very high in terms of quality (1.6 mean of answer scale), presentation (1.9) and clinical applicability (1.7) of the findings. The component of presentation of the findings, however, had the lowest level of agreement in this category. In the free text of the questionnaire, participants noted that the presentation may be improved by emphasizing positive findings (e.g., pop-up function in the electronic patient chart) or by offering the option of presenting cumulative findings. The practical application of the results in terms of interpreting antibiograms or identifying first-line antiinfectives was also highlighted as a need for improvement (data not shown).

## Discussion

Our point prevalence study provided valuable insights into the need to include microbiological components in the evaluation of an established AMS structure, which in our university hospital covers all clinical areas with more than 2500 documented—mostly telephone—consultations per year. Only by the additional assessment of pre- and post-analytical microbiologic features beyond classical AMS aspects, significant gaps in the AMS system could be identified. A clear lack of awareness regarding the planning of microbiological diagnostic as well as the implementation of laboratory findings in further therapy strategies could be shown.

On the day of the PPS antimicrobial agents were administered in 24.4% of the patients. This matches with other AMS data across Europe and North America, where administration rates between 27.4 and 38.6% were demonstrated [8, 9]. Considering the predefined quality criteria, 31.7% of cases were classified as inadequate, with lack of microbiological awareness being the largest failure category in this PPS (35.4% of cases).

With regard to the antimicrobials used, especially substances with broad to very broad activity were shown to be inappropriately used. This was particularly evident with meropenem, which accounted for only 7.7% of all antimicrobials used, but was administered inadequately in 60% of all cases. Lack of microbiological awareness here clearly emerged as the main cause for an inadequate treatment (53.3%). Remarkably, comparable results were observed for amoxicillin/clavulate (50.0%), piperacillin/tazobactam (44.4%), clindamycin (42.7%) and ceftriaxone (28.6%). Our data suggest that, especially with broad- and very broad-spectrum antiinfectives, there is considerable potential to improve the rational administration of substances by enhancing microbiological sensitization at both pre- and post-analytical levels. In contrast, inadequate utilisation of cefazolin and cefuroxime were mainly related to dosage failures or improper duration of therapy, especially the latter was associated with inadequately prolonged perioperative prophylaxis.

Knowledge of the relevant infectious entity is essential to address pre- and post-analytical problem areas from a clinical microbiology perspective [10]. In this regard, our study was able to highlight perioperative prophylaxis (44.4% of cases), followed by urinary tract infections (42.9%), pneumonia (34.1%), and skin/soft tissue infections (36.7%) as the main areas of inadequate antimicrobial use. In these entities, high rates of pre- and post-analytical failures could be demonstrated, as can be seen in the example of urinary tract infections, where, in eight out of nine patients with UTI (88.9%) no implementation of available microbiologic results was found. Similar results were observed in skin and soft tissue infections and pneumonia, where microbiological results were neglected in four out of six cases (66.7%) and three out of six patients (50%), respectively.

This is where a more diagnostic view, even within an established AMS structure, can make a valuable contribution. Morado et al. address this issue in a recent review [6] by demonstrating that implementation of pre- and post-analytical improvement as well as implementation of diagnostic stewardship measures can lead to a significant reduction of inadequate diagnostic ordering, but also of the total amount of administered antiinfectives. It suggests a high potential for well-targeted DSS interventions, particularly in the post-analytical segment, with clinicians guided by microbiological results that are clinically focused.

In our clinic the benefits of weekly direct support from a member of the AMS team on the ward were impressively demonstrated in patients with prosthetic joint infections. With only 3 out of 14 cases rated as inadequate (21.4%), this complex infectious entity was the second most acceptable in terms of appropriate antimicrobial treatment. However, this beneficial effect of on-site infectious disease consultation could not be demonstrated in all places where a weekly visit is scheduled: in our clinic two out of three intensive care units (the ICU in the Department of Cardiology and one of two ICUs in the Department of Anesthesiology) receive weekly infectious disease consultation as part of a clinical ward round by the AMS team. In the cardiology ICU, all six patients receiving antimicrobial therapy at the time of the PPS had critical deviations in terms of either inadequate dosing, lack of bacteriological sampling or lack of evaluation of the anti-infectives. On the other hand, opposite results were obtained at the anesthesiology ICUs. Although, only one of the two ICUs is visited as part of a weekly ward round, there was an equivalent proportion of adequate antimicrobial treatment in all qualities of 83.3% in both ICUs (5 out of 6 patients and 10 out of 12 patients, respectively; data not shown). One explanation for this could be the setting of the PPS, which only allows a momentary assessment of the situation. On the other hand, the small number of patients in some cases may have been the reason why the consultation effect was not equally detectable in all ICUs.

### Survey analysis

Interestingly, the questionnaire results did not allow any direct conclusions to be drawn about the substantial error rates on the ward. In fact, despite the very low response rate of only 7.1%, the majority of respondents were subjectively satisfied to very satisfied with the clinical applicability of the microbiological findings and regularly consulted the empirical treatment schemes provided by the AMS team. The authors suspect a relevant bias with respect to clinicians who responded to the questionnaire and the clinicians and their level of experience in daily practice, as 81.1% of the participating clinicians had advanced training (including 53.6% with 10 or more years of service) and could therefore be counted among the group of experienced doctors. However, the day-to-day work on the ward is mainly carried out by clinicians with far less clinical experience. This includes, in particular, ordering laboratory tests as well as the interpretation and evaluation of the resulting data.

### Diagnostic stewardship as a key to reducing inadequate administration of antiinfectives

The causes of non-observance of microbiologic results, which were found to be a major cause of inappropriate antiinfective use in the present study can be manifold and both our laboratory and the clinical environment are not unaffected. In the pre-analytical phase, weaknesses might already be found in the order-entry system if there is no guidance on appropriate sampling with respect to material, place of collection or shipping conditions. With regard to the analytical and post-analytical phase final cultural results are often not available until 48 h or later, which often leads to different ordering and receiving parties on the ward. This aspect might be exacerbated by increasing team rotation or incomplete documentation in the patient chart. Microbiologic results presented in a non-cumulative manner as well as a lack of differentiation between findings with and without pathogen detection (as currently in our case) represent additional weaknesses. The antibiogram is possibly overloaded with merely microbiologic information, especially in polymicrobial results, but does not allow clinical derivation of e.g., the most suitable antiinfective agent(s) and thus guidance of the clinician. This is where DSS can make a difference.

DSS was firstly coined by Morgan et al. with the concept of laboratories helping to increase the proper use of antimicrobials [5]. It aims to evaluate and improve laboratory-dependent processes of requesting, performing, and reporting diagnostic test results to consistently enhance diagnostic quality, optimize treatment, and improve patient outcome [11–14]. Thus microbiology as a core component of AMS strategies [2, 3, 7] is ideally suited to orchestrate this concept, provided that the laboratory fulfils its role as a key function. DSS consequently spans the range from pre-analytic and analytical processes to post-analytics (Table 7). Pre-analytical DSS strategies target the provision of adequate processes for a low-threshold information regarding adequate sample collection, request and shipment modalities (e.g., online database). Moreover, indication-triggered decision systems are an effective way to increase the accuracy of laboratory testing [2, 13, 15] as they are able to cause a significant reduction in both overdiagnosis and subsequently overtreatment for example concerning urinalysis or stool analysis [13, 16].

**Table 7:** Diagnostic stewardship aspects in microbiologic processes

Phase of microbiologic diagnostic	DSS aspects in microbiologic processes
Pre-analytic	Online catalogue or database of analytic spectrum including information on sample, sample container and shipment and clinical relevance of specific analysis IT-based order-entry systems allowing decisively specification of material and appropriate investigation Decision systems guiding the clinician and allowing target-oriented testing Training of target groups Evaluation
Analytic	Guideline—conform diagnostic and algorithms (e.g., EUCAST, CLSI) Highly automated laboratory processes with low turnaround time of rapid test systems QM-system and evaluation Training of laboratory staff Interdisciplinary communication between clinical and laboratory staff (trainings, meetings)
Post-analytic	Embedded comments and interpretation of results e.g.,: -- possible primary focus of microorganisms in blood cultures -- notice of suspected contamination -- note on distinction with regard to infection vs. colonization Nudging of clinical implications Reporting of physiological flora instead of single microorganisms (where clinically possible) Selective reporting of microorganisms Selective reporting of antimicrobial agents Cascade reporting of antimicrobial agents Labeling 1st line antimicrobial agents (related to the underlying infection) Training of target groups Evaluation

*EUCAST* European Committee on Antimicrobial Susceptibility Testing, *CLSI* Clinical and Laboratory Standard Institute

In the analytical phase of microbiologic diagnostic the laboratory is responsible for providing state-of-the-art diagnostics in terms of methodology and technical equipment, sample processing, and rapid transmission of findings. This requires a highly skilled team of laboratory technicians and microbiologists who are able to identify relevant correlations and detect pathogens and resistance patterns in a clinically oriented manner to provide targeted laboratory results for clinicians—ways that we have been putting into practice in our laboratory for years.

In the post-analytic phase, providing clinically applicable results is the key aspect. The findings should support or pave the way for therapeutic and further diagnostic steps or interventions by clinicians [3, 14, 15]. Possible ways for the laboratory are the embedding of comments, the labeling of first-choice antiinfectives in the antibiogram in relation to the clinical diagnosis, and selective reporting of substances. The latter has only been used rudimentarily in our hospital to date and only refers to the antibiogram of *Pseudomonas aeruginosa*, in which meropenem tested as sensitive is only indicated if there is resistance to ceftazidime or cefepime or if the use of meropenem is indicated as part of the current therapy. This is intended to prevent excessive use of the substance with the remainig beta-lactam antibiotics tested as “I” (intermediate).

Interestingly the subsuming of pathogens from physiological flora instead of a polymicrobial result might prevent the inappropriate use of anti-infectives. This process, known as “nudging,” are interventions that allow clinicians to elicit decisions through appropriate low-threshold decision architecture without being manipulative [13, 17]. Conversely, even indicating the lack of pathogens can be useful as Musgrove et al. demonstrated in their study in 2018, where stating of the absence of MRSA or *Pseudomonas aeruginosa* in respiratory secretions led to de-escalation of broad antibiotic therapy against these pathogens [18]. While comments on results alone might have little potential for improvement or may even be counterproductive [19], a clear interpretative annotation of a specific parameter constellation (e.g., *Clostridioides difficile* diagnosis) can significantly reduce the number of antibiotic therapies [20].

Skillful microbiological guidance of the clinician through cascade reporting can further contribute to a more rational antimicrobial use especially given the considerable proportion of doctors with a low level of training on the ward. Here, antiinfectives with a narrow spectrum of activity are listed, while broader-acting substances are only included on the report if resistance to primary substances is seen. Vissichelli et al. demonstrated only recently that this approach led to a relevant decrease in the use ciprofloxacin and meropenem. Nevertheless, the authors state that exact planning of the interventions and precise coordination between the microbiology laboratory, the AMS team, ID consultants, and clinicians is necessary to successfully implement this project and to achieve a long-lasting effect [21].

However, a game changer in advancing such innovations could be the establishment of a dissemination and implementation (D&I) system that translates evidence-based changes and implications into a real-life situation [22]. D&I research is largely unknown, or at least unused, in everyday laboratory settings. However, it also provides an opportunity to demonstrate the success of AMS and DSS strategies to hospital leadership and stakeholders, ensuring continuity and advancement of these efforts.

Our study was able to identify a lack of microbiological awareness on the ward as a major cause of inadequate anti-infective use. Still, there are limitations to our survey that should be noted. First, the study is designed as a point prevalence study providing only a snapshot of the AMS situation, thus important strategies such as i.v./p.o. changes, pharmacokinetic measurements or TDM could not be captured. Second, this study is not suitable to analyze long-term aspects such as treatment outcome. Third, the focus of this study is certainly on the adult and elderly population, whereas neonates, children, and adolescents are by their nature only subgroups. Regarding the lack of microbiological awareness, there are also numerous limitations in the area of diagnostic stewardship as post-analytical DSS aspects, in particular, are currently performed only in a basic fashion by our laboratory. In contrast to clinical-chemical findings, our microbiological findings, which are visible by the clinicians within the electronic patient chart as a “pdf document” currently cannot be validated as “seen” by the ward physician. This means, that there is no control on the ward as to whether the findings have already been viewed and by whom.

Furthermore, our microbiological findings are currently not displayed cumulatively and must be opened individually by the ward doctors. The physicians are used to consult the cumulative view of laboratory parameters for a better presentation and over-view in which the microbiology results are not integrated at the moment. There is therefore a significant probability that findings might be overlooked. Furthermore, positive microbiological findings are not highlighted in the electronic patient chart, and therapy-guiding comments are only rudimentarily performed. This may contribute substantially to the lack of clinical implication of the results and indicates a high optimization potential in both the pre- as well as the post-analytical phase of microbiologic results delivery and calls for the need to strengthen DSS measures in our hospital.

The survey of physicians, which also covered their level of training, showed how complex the problem of the post-analytical implication of microbiological findings is. We did not qualitatively record the level of training, with regard to infectious disease aspects in the PPS. However, the results from the questionnaire strongly suggest that there is a considerable discrepancy between the participants in the survey, who are predominantly highly specialized physicians, and the team of physicians actually working on the ward. The conclusions that can be drawn from the assessment of the level of training provide clear indications that distinct improvement measures such as selctive reporting or cascade reporting of findings are required in our clinic to raise awareness of microbiological findings and make them more clinically applicable. Doctors with a low level of training need to be addressed more specifically in the training courses offered by the AMS team.

## Conclusion

In summary, we were able to show that in our PPS, the main reason for an overall inadequate antimicrobial therapy was lack of microbiological awareness with regard to the absence of initial specimen collection and the lack of implementation of microbiological results in further treatment management. This highlights that, in addition to classical AMS issues, pre- and post-analytical aspects in particular need to be addressed in the context of DSS considerations. Only in this way is it feasible to gain a holistic view at the whole stewardship process. The microbiology laboratory has a key role to play here, as it can influence pre- and post-analytical processes in addition to its original diagnostic tasks, and thus play a decisive role in shaping DSS—as long as it is aware of its central role in the setting of infectious diseases. Skillfully chosen DSS measures by the microbiology laboratory could therefore lead to improvements in the overall use of antiinfectives, and other clinical microbiology laboratories should be encouraged to become aware of this important role in the context of infectious disease management. This study has highlighted areas that are of great importance also to our laboratory and the AMS team, which we will be exploring in more detail in the future, particularly to improve the post-analytical phase of microbiological infection diagnostic.

## Data Availability

All data used and analyzed in this study are available from the corresponding author upon reasonable request.
